# COVID-19 vaccination coverage among adolescents aged 12–17 years in three provinces of eastern China: A cross-sectional survey, 2021

**DOI:** 10.3389/fpubh.2022.919190

**Published:** 2022-07-22

**Authors:** Taishun Li, Ruowen Qi, Bingwei Chen, Yuqian Luo, Wenjun Zhang, Yi-Hua Zhou, Biyun Xu

**Affiliations:** ^1^Medical Statistics and Analysis Center, Nanjing Drum Tower Hospital, Nanjing University Medical School, Nanjing, China; ^2^Department of Biostatistics, School of Public Health, Southeast University, Nanjing, China; ^3^Department of Laboratory Medicine and Biomedicine Statistics, Nanjing Drum Tower Hospital, Nanjing University Medical School, Nanjing, China

**Keywords:** COVID-19 vaccine, cross-sectional, vaccination rate, adolescent, China

## Abstract

High vaccination coverage is essential to prevent and control the spread of the COVID-19 epidemic. Currently, the real-world acceptance of COVID-19 vaccines among adolescents aged 12–17 years in China has not been reported. We aimed to assess the acceptance rate of COVID-19 vaccination among adolescents in eastern China and to identify factors associated with the intention to get vaccinated against COVID-19. We conduct a cross-sectional questionnaire survey among adolescents from three provinces in the eastern part of China from 16 August to 28 October 2021. The questionnaires were distributed to 2,100 students, and 2,048 students completed the questionnaires. The results showed that 98.4% (2,016/2,048) of adolescents had received at least one dose of the COVID-19 vaccine and 1.6% (32/2,048) declined the vaccination. The participants from rural districts, or whose parents were vaccinated, were more likely to accept the vaccine. The main reason for declining vaccination was worry about vaccine safety (25%). The main adverse event after the vaccination was pain at the injection site. In conclusion, the vaccine coverage rate reached 98.4% among the adolescents in this study, which met the criteria for herd immunity to severe acute respiratory syndrome coronavirus 2 (SARS-CoV-2). The high vaccination rate is beneficial to the prevention and control of the COVID-19 pandemic.

## Introduction

First occurred at the end of 2019, the COVID-19 caused by severe acute respiratory syndrome coronavirus 2 (SARS-CoV-2), has caused a pandemic by March 2020 (1). The disease has spread worldwide, instigating a pandemic causing more than 533 million confirmed cases and leading to more than 6.3 million deaths worldwide based on the WHO weekly epidemiological update as of June 16, 2022 (2). The pandemic of COVID-19 has become a major threat to global public health, resulting in a global economic downturn and social panic (3). In addition, the pandemic has disrupted some normal medical activities such as pregnancy and childbirth (4–6). In response to the serious global impacts of COVID-19, the vaccine has become one of the most effective strategies for ending the pandemic (7, 8). There is evidence that the vaccine can not only provide direct immunity to the vaccinated individuals but also reduce infections among the unvaccinated individuals by building herd immunity from large-scale coverage of the population with immunity (9).

China has developed various COVID-19 vaccines, mainly composed of inactivated SARS-CoV-2 (10). The efficacy and safety of the vaccines have been investigated in phase 3 clinical trials (11). The vaccines have been widely used in adults in China since December 2020, and the inactivated COVID-19 vaccines have also been approved for WHO emergency use listing (12). Recently, studies showed that COVID-19 vaccination alleviated the disease severity caused by wild-type as well as variants of SARS-CoV-2 (13, 14). At the initial phase of vaccination against COVID-19, the vaccines were exclusively applied to adults (≥ 18 years old) peoples, since the disease causes severe health impacts in adults with relatively high mortality, yet it is relatively mild in adolescents and children (15). However, with the recent global COVID-19 pandemic, especially after the delta variant had become the main transmitted strain, an increasing number of infection events reminded people to be vigilant about the spread of COVID-19 among children and adolescents (15, 16). Since July 2021, the vaccination for children and adolescents has been implemented in China, and the vaccines administered for adolescents are composed of inactivated SARS-CoV-2 (Sinovac Coronavac or Sinopharm BBIBP-CorV, China).

Adolescents aged 12–17 years are of school age, and the coverage of vaccination is essential to prevent and control the spread of SARS-CoV-2 in schools. The success of herd immunity will depend on the vaccination rate. However, numerous factors could affect the vaccination rate for children and adolescents during pandemics, such as parents' opinions, school policies, consideration of vaccine safety, and others (17, 18). Hence, it is crucial to know the true vaccination rate among adolescents and to explore the associated factors with the acceptability of the COVID-19 vaccine.

The objective of this study was to assess the actual acceptance rate of the COVID-19 vaccine among adolescents in three provinces of eastern China and to identify factors associated with the intention to get vaccinated against COVID-19 in children and adolescents. Understanding the actual vaccination rate and the factors associated with the vaccination intention among adolescents may support government and public health officials to find better ways to persuade people to get vaccinated and finally reach herd immunity to SARS-CoV-2.

## Materials and methods

### Subjects and procedures

We performed a cross-sectional survey, from 16 August to 28 October 2021, on the real-world acceptance of COVID-19 vaccination among adolescents in the eastern region of China. Since high school students in China are usually aged 12–17 years, we distributed the questionnaires among the students in high schools. Based on the economic situation, the six provinces in the eastern part of China can be generally divided into high (Jiangsu and Zhejiang), middle (Shandong and Fujian), and low (Anhui and Jiangxi) levels of economic development. We selected one province from each level, namely Jiangsu, Fujian, and Anhui. In each province, we selected one school from urban areas and one school from rural areas. Students were selected using a cluster sampling method, and once their schools were included in the study, all students were given questionnaires. The students who participated in this survey were informed at the beginning of the questionnaires, as the statement “If you are willing to participate in this survey, this means that you like to give informed consent” is presented at the top of the questionnaire form (see Supplementary File 1). The parents/legal guardians received notices about the survey before it started. The protocol was approved by the Ethics Committee of the Nanjing Drum Tower Hospital (2021-462-01).

### Survey design and pre-testing

The questionnaire was formulated based on the previous reports (17–24) and the opinion of experts in infectious diseases. Before the actual survey, we conducted a small-scale pre-survey on the target population and modified the survey questions and layout format after the feedback from the participants.

### Measures

The questionnaire covered: (1) demographic characteristics, (2) attitudes toward vaccine, (3) acceptance of COVID-19 vaccine, and (4) adverse events after vaccination. The survey objective was to understand adolescents' opinions on the COVID-19 vaccine and the actual vaccination rate. There was one question designed to calculate the acceptance rate of the COVID-19 vaccine: We asked the respondents to answer the question “Have you accepted the COVID-19 vaccine?” followed by an open-ended question “Why?” or “Why not?,” with a free text box. An additional text file shows the questionnaire in more detail (see Supplementary File).

### Data analysis

Basic descriptive statistics and frequencies were used to describe all variables. Univariate analysis was conducted to determine factors associated with vaccination acceptance: Mann-Whitney test for comparing non-normal continuous variables, independent *t*-test for comparing normally distributed continuous variables, and Chi-square test or Fisher's exact test for categorical variables. Multivariate logistic regression analysis was also used to estimate the adjusted odds ratio (OR) of vaccination, using all the variables that showed significance (*p* < 0.05) in the univariate analysis. All analyses were conducted with R version 4.04. *p* ≤ 0.05 was considered statistically significant.

## Results

### Characteristics of participants

**Figure 1:**
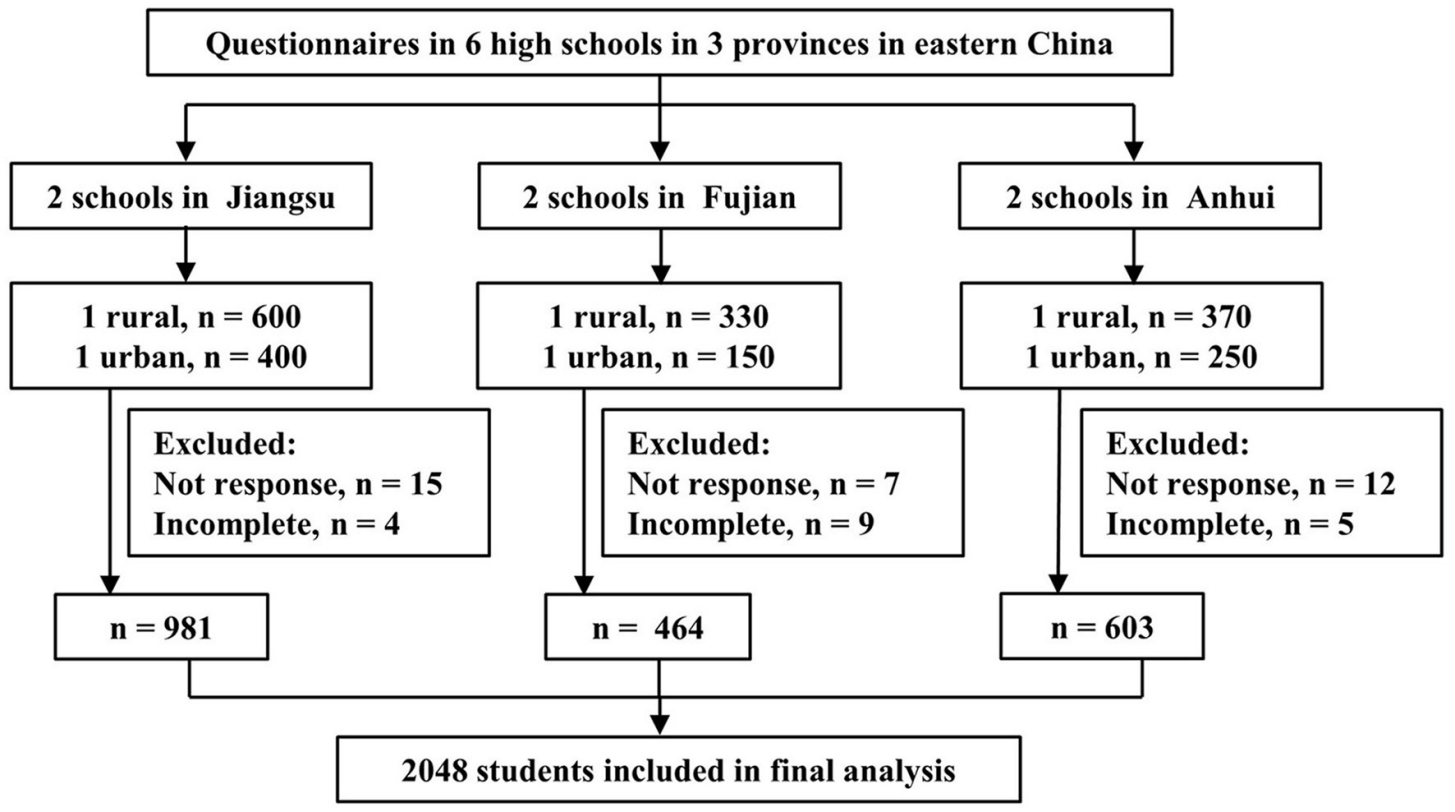
Flow chart of selection of the study participants.

The questionnaires were distributed to 2,100 target students, and a total of 2,048 (97.5%) responded. Figure 1 shows the flow chart of study participants. All results are analyzed based on the 2,048 participants who completed the survey questionnaire. The mean age was 14.5 years (range 12–17 years), more than half (53.4%) were aged 15–17 years, and 48.8% of them were men. Most participants (60.5%) were from rural areas, and 43.5% of participants were in senior high school. The demographic information of participants is presented in Table 1.

**Table 1:** Participant characteristics (*n* = 2,048).

**Characteristic**	
**Age, y (*n* = 2,048)^*a*^**	
Mean ± SD	14.55 ± 1.5
Range	12–17
**Characteristic**	***n* (%)**
Gender (*n* = 2,045)	
Female	1,047 (51.2)
Male	998 (48.8)
Province (*n* = 2,048)	
Jiangsu	972 (47.5)
Anhui	603 (29.4)
Fujian	473 (23.1)
Race (*n* = 2,045)	
Han	1,995 (97.6)
other	50 (2.4)
District (*n* = 2,020)	
Urban	797 (39.5)
Rural	1,223 (60.5)
Education school (*n* = 2,048)	
Junior high school	1,157 (56.5)
Senior high school	891 (43.5)

a *Unless otherwise indicated, data are the number (percentage) of survey respondents*.

Overall, 2,016 (98.4%) participants accepted the COVID-19 vaccine, and 32 (1.6%) participants did not. Of the 2,016 vaccinated participants, 1,918 (95.1%) completed the second dose, and 98 (4.9%) did not receive the second dose because the time intervals were <2 weeks. The results of the univariate analysis between the vaccinated and unvaccinated groups were summarized in Table 2. The results showed that the acceptance of COVID-19 was associated with the district, willingness for vaccination, and whether the parents were vaccinated. Table 3 shows the results of multivariable binary logistic regression analysis on the factors associated with vaccine acceptance. The results indicated that the participants from rural districts or parents who were vaccinated were more likely to accept the vaccine.

**Table 2:** Univariate analysis for factors associated with COVID-19 vaccine acceptance.

	**Not vaccinated**		**Vaccinated**		***p***
	***n***	**%**	***n***	**%**	
Overall	32	1.6	2,016	98.4	
Age group (year)					0.070
12–14	20	62.5	935	46.4	
15–17	12	37.5	1,081	53.6	
Gender					0.826
Male	15	46.9	983	48.8	
Female	17	53.1	1,030	51.2	
District					0.007
Rural	12	37.5	1,211	60.9	
Urban	20	62.5	777	39.1	
Worry about vaccine safety					0.131
Worry	8	25.8	311	15.5	
No worry	23	74.2	1,702	84.5	
Confidence for vaccine effectiveness					0.668
Confident	29	93.6	1,913	95.0	
No confidence	2	6.4	101	5.0	
Willingness for vaccination					0.001
Yes	27	87.1	1,954	97.1	
No	4	12.9	59	2.9	
Medical workers in the family					0.240
Direct relatives	4	12.9	158	8.0	
Other relatives	9	29.0	399	20.2	
No	18	58.1	1,414	71.8	
Someone in the family participates in COVID-19 prevention and control work					0.920
Yes	6	19.4	374	18.6	
No	25	80.6	1,632	81.4	
Whether the parents are vaccinated					0.003
Both vaccinated	24	77.4	1,868	92.8	
Neither vaccinated	2	6.5	13	0.7	
One of two vaccinated	5	16.1	132	6.5	
Is life affected by the COVID-19					0.151
Seriously affected	7	22.6	265	13.2	
Affected but not serious	11	35.5	1,025	50.9	
No effect	13	41.9	722	35.9	

**Table 3:** Factors associated with COVID-19 vaccine acceptance expressed with odds ratio, in multivariate binary logistic regression.

**Factors**	β	**SE**	**OR (95% CI)**
District			
Rural	Ref	-	-
Urban	−0.85	0.39	0.43 (0.20–0.92)
Willingness for vaccination			
No	Ref	-	-
Yes	1.14	0.58	3.12 (1.01–9.66)^*^
Whether the parents are vaccinated			
Neither vaccinated	Ref	-	-
Both vaccinated	2.09	0.81	8.10 (1.67–39.35)^*^
One of two vaccinated	1.38	0.90	3.96 (0.68–23.10)

* *p < 0.05*.

### Reasons for acceptance or decline of the COVID-19 vaccination

The detailed reasons for acceptance or decline of COVID-19 vaccination are presented in Table 4. The results showed that 70.6% of the vaccinated participants took the vaccine based on their own decision without a specific reason. The two other main reasons for acceptance of the COVID-19 vaccination were requested by the school manager (14.6%) and encouraged by family members (11.6%). On the other hand, worry about vaccine safety accounted for 25% (8/32) of those who declined the vaccination.

**Table 4:** Reasons for acceptance or decline of COVID-19 vaccination in participants.

**Reasons**	***n*** **(%)**
Acceptance of COVID-19 vaccination (*n* = 2,016)	
Totally own decision, no specific reason	1,411 (70.6)
Consulted the scientific literature	216 (10.8)
Encouraged by family members	231 (11.6)
Encouraged by teachers	210 (10.5)
Encouraged by friends	104 (5.2)
Encouraged by doctors	57 (2.9)
Encouraged by propaganda News	101 (5.1)
Requested by School manager	291 (14.6)
Unwilling to, but followed school arrangement	60 (3.0)
Others	130 (6.5)
Decline the COVID-19 vaccination (n=32)	
Not willing to without reason	4 (13.3)
Not willing to, but with the excuse such as toothache or cold	1 (3.3)
Worried about the efficacy of the vaccine	2 (6.7)
Worry about the short-term protection (3–6 months)	2 (6.7)
Worry about the vaccine safety	1 (3.3)
Worry about political involvement	2 (6.7)
Influenced by parents or relatives	1 (3.3)
Influenced by friends or teachers	0 (0.0)
Influenced by Internet	0 (0.0)
Worry about the safety without own chronic diseases	2 (6.7)
Worry about the safety because of own chronic diseases	5 (16.7)
Willing to take, but declined by vaccination staff	6 (20.0)
Others	3 (9.3)

### Self-reported adverse events in vaccinated participants

The self-reported adverse events in vaccinated participants are shown in Table 5. Of the 2016 vaccinated participants who completed the first vaccine dose, 433 (21.5%) reported one or more adverse events, with 0.8% (17/2,016) adverse events requiring treatment and 1.4% (29/2,016) adverse events affecting the study and life. Of the 1,918 vaccinated participants who accepted the second vaccine dose, 396 (20.6%) had adverse events. Table 6 presents the 10 common adverse events in the vaccinated participants, and the most common adverse event after either the first or second dose was pain at the injection site.

**Table 5:** Self-reported Adverse events in vaccinated participants.

**Variables**	**Dose 1**, ***n*** **(%)**	**Dose 2**, ***n*** **(%)**	**Both**, ***n*** **(%)**
Self-reported Adverse events			
Completed the first dose (*n* = 2,016)	433 (21.5)	-	-
Completed the second dose (*n* = 1,918)	419 (21.9)	396 (20.7)	284 (14.8)
Adverse events requiring treatment			
Completed the first dose (*n* = 2,016)	17 (0.8)		
Completed the second dose (*n* = 1,918)	15 (0.8)	13 (0.7)	6 (0.3)
Adverse events affecting life			
Completed the first dose (*n* = 2,016)	29 (1.4)		
Completed the second dose (*n* = 1,918)	26 (1.4)	23 (1.2)	12 (0.6)

**Table 6:** Top 10 incidences of adverse events after vaccination.

**Self-reported side effects**	***n*** **(%)**
After the first dose (*n* = 2,016)	
Pain at the injection site	281 (13.9)
Local stabbing pain in the arm	93 (4.6)
Redness and swelling at the injection site	58 (2.9)
Feeling weak after injection	57 (2.8)
Delayed Menstruation (only for female)	41 (4.0)
Insomnia	40 (2.0)
Dizziness	38 (1.9)
Loss of appetite	37 (1.8)
Headache	36 (1.8)
Early menstruation (only for female)	29 (2.8)
After the second dose (*n* = 1,918)	
Pain at the injection site	273 (14.2)
Local stabbing pain in the arm	82 (4.3)
Redness and swelling at the injection site	48 (2.5)
Feeling weak after injection	45 (2.4)
Loss of appetite	36 (1.9)
Dizziness	34 (1.8)
Delayed Menstruation (only for female)	34 (3.4)
Early menstruation (only for female)	33 (3.3)
Headache	31 (1.6)
Insomnia	30 (1.6)

## Discussion

In this cross-sectional study among adolescents between 12 and 17 years in three provinces of the eastern part of China, the majority (98.5%) of adolescents had received at least one dose of the COVID-19 vaccine, and 93.4% of adolescents took the second dose. The vaccination rate in teenagers is higher than that (70.1–86.2%) in adults in China (25–27), and that (42.4%) in adolescents in the USA (28). Independent factors associated with high uptake included the rural district, willingness for vaccination, and parents who were vaccinated.

The results showed that the vaccination rate in students is very high (98.5%), indicating that the school requirement of vaccination before school enrolling helps to increase the vaccination rate. Adolescents aged 12–17 years are in junior or senior high school age, and learning is the most important part of their life. However, their study life was once severely disrupted due to the epidemic of COVID-19. Higher levels of anxiety were reported in adolescents who had concerns about the quality of their education because of long-term online learning (17). Hence, adolescents were more likely to follow the school regulations to accept the COVID-19 vaccination.

Our study reflected that the adolescents whose parents got vaccinated were more likely to accept the vaccine. This demonstrated that parents' attitudes and behaviors have a great influence on adolescents' willingness to receive the vaccine against COVID-19 (29), which highlights the need for publicizing parents about COVID-19 vaccination for increasing the willingness of adolescents in the future. The results showed that the adolescents living in rural areas had a higher vaccination rate, compared with those living in urban areas. This could be the reason that the participants in cities were concerned more about the vaccine safety and effectiveness because they were exposed to different information about the COVID-19 vaccination from various sources, which gave them more uncertainty about the vaccination.

For increasing the vaccination rate, two of the six schools in our survey directly set up vaccination stations for COVID-19 in the school. This method has many advantages for the COVID-19 vaccination campaign. On the one hand, centralized vaccination on campus facilitates students and increases their enthusiasm for vaccination. Students who are initially afraid of vaccination may dispel their concerns when they are encouraged by classmates and teachers on-site, which improves the vaccination rates. On the other hand, the concentrated medical observation after vaccination is conducive to discovering the adverse events of the vaccine in time and can make a better assessment of the safety of the vaccine in adolescents. Previous studies also indicated that vaccine administration on-site at schools is an effective method to improve adolescent vaccination rates (28).

The main reason for vaccine hesitancy among unvaccinated participants included the concern about vaccine safety and effectiveness. Our results are in agreement with the findings in the vaccination intention surveys, in which the most critical reason for the decline of the COVID-19 vaccination is the concern for the safety and effectiveness of the vaccine (30–34). This highlights the need for the government to take measures to publicize the effectiveness and safety of vaccines. In this study, the self-reported adverse events occurred in 21.5% of vaccinated participants after the first dose and 20.6% after the second dose, lower than that reported in adults (35–37). Moreover, the vast majority of subjects with adverse events just had mild adverse events that did not affect their life and did not require treatment. Thus, this study provided evidence that inactivated COVID-19 vaccines are highly safe in adolescents.

There are some limitations in this study. First, we only investigated students and did not survey the individuals of the same age who did not go to school; thus, it remains unclear if our findings are generalizable to general adolescents. However, the school enrollment rates of junior and senior high schools in the three provinces are all above 90% (38). Second, all the adverse events were self-reported and not real-time monitored. The survey subjects were all minors and had a low level of awareness of the adverse events in the questionnaire, which might be subject to reporting bias. Hence, the name of the proprietary adverse event was explained in a popular way in the questionnaire to facilitate the understanding of the respondents. Third, since this study only evaluated the vaccination rates during the first 2 months of the program, the vaccination coverage might have increased with the popularization of the COVID-19 vaccination. Thus, long-term follow-up surveys are required to estimate the change in actual acceptance of the COVID-19 vaccine in adolescents. Fourth, the acceptance of vaccination in this study might be influenced by the enforced regulation in the schools and this could be different in different schools. Lastly, our study only selected three provinces in the eastern part of China, and only two schools were selected in each province, which might have some selection bias.

## Data availability statement

The raw data supporting the conclusions of this article will be made available by the authors, without undue reservation.

## Ethics statement

The studies involving human participants were reviewed and approved by Ethics Committee of the Nanjing Drum Tower Hospital. Written informed consent from the participants' legal guardian/next of kin was not required to participate in this study in accordance with the national legislation and the institutional requirements.

## Author contributions

Conceptualization: TL, RQ, BX, and Y-HZ. Methodology and resources: BX and TL. Validation: BX, BC, and TL. Formal analysis: RQ. Investigation: BX, TL, RQ, YL, and WZ. Data curation: TL and RQ. Writing—original draft preparation: TL. Writing—review and editing, project administration, and supervision: BX and Y-HZ. All authors contributed to the article and approved the submitted version.

## Funding

This research was funded by Jiangsu Innovative and Entrepreneurial Talent Programme (Grant Number: JSSCBS20211510).

## Conflict of interest

The authors declare that the research was conducted in the absence of any commercial or financial relationships that could be construed as a potential conflict of interest.

## Publisher's note

All claims expressed in this article are solely those of the authors and do not necessarily represent those of their affiliated organizations, or those of the publisher, the editors and the reviewers. Any product that may be evaluated in this article, or claim that may be made by its manufacturer, is not guaranteed or endorsed by the publisher.

## Supplementary material

Supplementary File 1 A questionnaire on COVID-19 vaccination among adolescents aged 12 to 17 in three provinces: Anhui, Jiangsu, and Fujian, China, 2021.
