# Supplementary material for: COVID-19 vaccination coverage among adolescents aged 12–17 years in three provinces of eastern China: A cross-sectional survey, 2021
Source: Front Public Health. 2022 Jul 22;10:919190. doi: 10.3389/fpubh.2022.919190 (PMC9355633; doi:10.3389/fpubh.2022.919190)
Supplement: Supplementary File 1 — A questionnaire on COVID-19 vaccination among adolescents aged 12 to 17 in three provinces: Anhui, Jiangsu, and Fujian, China, 2021. [file Data_Sheet_1.pdf]

# A questionnaire on COVID-19 vaccination among adolescents aged 12 to 17 in three provinces: Anhui, Jiangsu and Fujian, China, 2021

This survey aims to investigate the real-world acceptance of COVID-19 vaccine and related issues among adolescents aged 12 to 17 in China. The questionnaire is anonymous. Whether or not answering to the questionnaire is all determined based on your own willingness, and will not influence any of your interests and will not cause any negative effect to you. If you are willing to participate in this survey, this means that you like to give informed consent. Please complete the questionnaire independently and objectively. Thank you for your support and cooperation.

## I. Participant Characteristics

**Q1:** You are from [**Single choice**]: _______ Province
A. Anhui B. Jiangsu C. Fujian

**Q2:** Nationality [**Single choice**]: _______.
A. Han B. Other

**Q3:** Registered Residence [**Single choice**]: _______.
A. Rural area B. City

**Q4:** Gender [**Single choice**]: _______.
A. Male B. Female

**Q5:** Your birthday [**Blank filling**]: ______year ______month ______day

**Q6:** Your grade [**Single choice**]: _______.
A. Junior high school B. High school

**Q7:** Height [**Blank filling**]: ______cm

**Q8:** Weight [**Blank filling**]: ______kg

## II. About the general knowledge of COVID-19 vaccine

**Q9.** Currently, what kind of vaccine is the most vaccinated in China **[Single choice]: ______**

A. No idea

B. Inactivated virus vaccines

C. Adenovirus recombinant vaccine

D. Recombinant coronavirus protein subunit vaccine

E. Coronavirus mRNA vaccine

**Q10.** Do you wish to gain knowledge about COVID-19 vaccine **[Single choice]: ______**

A. Willing B. Unwilling

**Q11.** Your knowledge source about COVID-19 vaccine **[Single or multiple choices]: ______**

A. Various news media

B. Introduction from parents or relatives

C. Introduction from physicians

D. Expert lectures in school

E. Other

F. Little knowledge about coronavirus vaccine

**Q12.** Your concern about the safety of COVID-19 vaccine **[Single choice]: ______**

A. Worry B. Not worry

**Q13.** Your confidence about the efficacy of COVID-19 vaccine **[Single choice]: ______**

A. Confident B. Not confident

**III. About the intention of vaccination**

**Q14.** Are you willing to take COVID-19 vaccine **[Single choice]: ______**

A. Willing B. Unwilling

**Q15.** Did you ever hear about any side effect after acceptance of COVID-19 vaccine **[Single choice]: ______**

A. I've heard of B. I've never heard of

**Q16.** Are there any medical and health care professionals in your family **[Single choice]: ______**

A. In direct relatives

B. In other relatives

C. No

**Q17.** Did your family members participate in the COVID-19 anti-epidemic prevention work (e.g. medical staff, community worker, volunteer) **[Single choice]: ______**

A. Yes B. No

**Q18.** Did your parents get COVID-19 vaccine **[Single choice]: ______**

A. Both vaccinated

B. Neither vaccinated

C. Either vaccinated

**Q19.** Does your family support you to get vaccinated **[Single choice]: ______**

A. Support

B. Neutral

C. Not support

D. Unknown

**Q20.** Was your life affected by the COVID-19 epidemic **[Single choice]: ______**

A. Seriously affected

B. Affected

C. No effect

**Q21.** Have you been centrally isolated or isolated at home due to close contact with COVID-19 patients in the past 3 months **[Single choice]: ______**

A. Yes B. No

**IV. Real-world situation about the COVID-19 vaccination**

**Q22.** Did you receive the first dose of COVID-19 vaccine **[Single choice]: ______**

A. No B. Yes

If you received the first dose of COVID-19 vaccine, please answer the questions 23, 24. If you did not receive the first dose, please answer from question 50.

**Q23.** The reason for your acceptance of COVID-19 vaccination **[Single choice]: ______**

A. Based on own decision and no specific reason required

B. Hesitant initially, but vaccinated after consulting the scientific literature

C. Hesitant initially, but vaccinated after consulting family members (including immediate family members and cousins)

D. Hesitant initially, but vaccinated after consulting teachers (not including experts or professionals)

E. Hesitant initially, but vaccinated after consulting friends or classmates (not including experts or professionals)

F. Hesitant initially, but vaccinated after consulting a professional or an expert

G. Hesitant initially, but vaccinated after listen to news about COVID-19 vaccine

H. Hesitant initially, but vaccinated after the request by the school

I. Unwilling to get vaccinated, but had to be vaccinated at the request of the school

J. Other

**Q24.** Were there any adverse reactions after the first dose of vaccine? **[Single choice]: ______**

A. No  B. Yes

If you had adverse reactions after the first dose of COVID-19 vaccine, please answer the questions 25-34. If you did not have adverse reactions, please omit the questions 25-34, and answer question 35.

**Q25.** Local reactions **[Single or multiple choices]: _______**

A. Pain

B. Redness and swelling

C. Surrounding skin rash

D. Surrounding skin ulceration

E. Local tingling

F. Influence of limb function

**Q26.** If you got fever after injection **[Single choice]: _______**

A. <38.0 °C  B. 38.0-38.9 °C  C. >=39 °C

**Q27.** Influenza-like symptoms **[Single or multiple choices]: _______**

A. Fatigue  B. General pains and soreness

**Q28.** Headache or Dizziness **[Single or multiple choices]: _______**

A. Headache  B. Dizziness

**Q29.** Digestive symptoms **[Single or multiple choices]: _______**

A. Loss of appetite

B. Nausea

C. Vomiting

D. Abdominal pain

E. Diarrhea

**Q30.** Respiratory symptoms and sleep **[Single or multiple choices]: _______**

A. Insomnia

B. Cough

C. Sputum

D. Chest pain

E. Dyspnea

**Q31.** Menstrual disorders **[Single or multiple choices]: _______**

A. Earlier menses

B. Delayed menses

C. Heavy bleeding volume

D. Light bleeding volume

**Q32.** Anaphylactic reactions **[Single or multiple choices]: _________**

A. Pruritus

B. Rash

C. Urticaria

D. Larynx edema

E. Anaphylactic shock

**Q33.** Severity of adverse events **[Single choice]: _________**

A. Mild: requiring no treatment

B. Moderate: requiring treatment, including sick leave, but not hospitalization

C. Severe: requirement of hospitalization

**Q34.** Did adverse events affect your life or study **[Single choice]: _________**

A. Seriously affected

B. Affected

C. Little affected

D. No effect

**Q35.** Did you receive the second dose of COVID-19 vaccine **[Single choice]: ______**

A. No B. Yes

If you received the second dose of COVID-19 vaccine, please answer the question 36. If you did not receive the second dose, please omit the questions 36-46, and answer question 47.

**Q36.** Were there any adverse reactions after the second dose of vaccine? **[Single choice]: ______**

A. No B. Yes

If you had adverse reactions after the second dose of COVID-19 vaccine, please answer the questions 37-46. If you did not have adverse reactions, please omit the questions 37-46, and answer question 47.

**Q37.** Local reactions **[Single or multiple choices]: _______**

A. Pain

B. Redness and swelling

C. Surrounding skin rash

D. Surrounding skin ulceration

E. Local tingling

F. Influence of limb function

**Q38.** If you got fever after injection **[Single choice]: _______**

A. <38.0 °C B. 38.0-38.9 °C C. >=39 °C

**Q39.** Influenza-like symptoms **[Single or multiple choices]: _______**

A. Fatigue B. General pains and soreness

**Q40**. Headache or Dizziness **[Single or multiple choices]: _______**

A. Headache B. Dizziness

**Q41.** Digestive symptoms **[Single or multiple choices]: _______**

A. Loss of appetite

B. Nausea

C. Vomiting

D. Abdominal pain

E. Diarrhea

**Q42.** Respiratory symptoms and sleep **[Single or multiple choices]: _______**

A. Insomnia

B. Cough

C. Sputum

D. Chest pain

E. Dyspnea

**Q43.** Menstrual disorders **[Single or multiple choices]: _______**

A. Earlier menses

B. Delayed menses

C. Heavy bleeding volume

D. Light bleeding volume

**Q44.** Anaphylactic reactions **[Single or multiple choices]: _________**

A. Pruritus

B. Rash

C. Urticaria

D. Larynx edema

E. Anaphylactic shock

**Q45.** Severity of adverse events **[Single choice]: _________**

A. Mild: requiring no treatment

B. Moderate: requiring treatment, including sick leave, but not hospitalization

C. Severe: requirement of hospitalization

**Q46.** Did adverse events affect your life or study **[Single choice]: _________**

A. Seriously affected

B. Affected

C. Little affected

D. No effect

**Q47.** Do you think you will not infect COVID-19 after the vaccination **[Single choice]: _________**

A. Agree

B. Not agree

C. Don't know

**Q48.** Do you agree that the COVID-19 vaccine can reduce the risk of becoming severe after infection **[Single choice]: _________**

A. Agree

B. Not agree

C. Don't know

**Q49.** Do you agree to relax other preventive measures, such as wearing masks after vaccination **[Single choice]: _________**

A. Agree

B. Not agree

C. Don't know

If you did not accept any dose of vaccine, please answer questions 50-52, If you accepted at least one dose of vaccine, please answer from question 53.

**Q50.** The reasons for the decline of COVID-19 vaccine **[Single or multiple choices]: _________**

A. Unwilling to vaccinate for no reason

B. Unwilling to vaccinate, but find other excuses, such as catching cold, toothache, too busy to have time getting vaccinated, and others

C. Worry about the efficacy of vaccine, or ineffective

D. Worry about the short effective duration (only 3-6 months), and no long-term protection

E. Worry about the short period from research to application and being wait-and-see

F. Worry about the urgent approval due to political issue

G. Influenced by parents and relatives (not including experts or professionals)

H. Influenced by friends or teachers (not including experts or professionals)

I. Influenced by the information from Internet

J. Worry about the vaccine safety although in good health condition

K. Worry about the vaccine safety because of the presence of chronic diseases

L. Willing to be vaccinated, but declined by the vaccination staff

M. Other reason

**Q51.** Are you worried that you will infect COVID-19 in the future because you are not vaccinated **[Single choice]: _________**

A. Worry B. Not worry

**Q52.** Are you worried you will infect others if you infect COVID-19 because you are not vaccinated **[Single choice]: _________**

A. Worry B. Not worry

**V. Other questions**

**Q53.** Would you like to listen to the lecture about the COVID-19 vaccine **[Single choice]: _________**

A. Willing B. Unwilling

**Q54.** If there is a third booster of COVID-19 vaccine, are you willing to take it **[Single choice]: _________**

A. Willing B. Unwilling
